# 4-(4-Acetyl-5-methyl-1*H*-1,2,3-triazol-1-yl)benzo­nitrile: crystal structure and Hirshfeld surface analysis

**DOI:** 10.1107/S2056989018010885

**Published:** 2018-08-10

**Authors:** Julio Zukerman-Schpector, Cássio da S. Dias, Ricardo S. Schwab, Mukesh M. Jotani, Edward R. T. Tiekink

**Affiliations:** a Laboratório de Cristalografia, Esterodinâmica e Modelagem Molecular, Departamento de Química, Universidade Federal de São Carlos, 13565-905 São Carlos, SP, Brazil; b Departamento de Química, Universidade Federal de São Carlos, 13565-905 São Carlos, SP, Brazil; c Department of Physics, Bhavan’s Sheth R. A. College of Science, Ahmedabad, Gujarat 380001, India; d Research Centre for Crystalline Materials, School of Science and Technology, Sunway University, 47500 Bandar Sunway, Selangor Darul Ehsan, Malaysia

**Keywords:** crystal structure, 1,2,3-triazol-1-yl, nitrile, Hirshfeld surface analysis, NCI plots

## Abstract

The title compound, C_12_H_10_N_4_O, comprises a central 1,2,3-triazole ring (r.m.s. deviation = 0.0030 Å) flanked by N-bound 4-cyano­phenyl and C-bound acetyl groups, which make dihedral angles of 54.64 (5) and 6.8 (3)° with the five-membered ring, indicating a twisted mol­ecule. In the crystal, the three-dimensional architecture is sustained by carbonyl-C=O⋯π(triazo­yl), cyano-C≡N⋯π(triazo­yl) (these inter­actions are shown to be attractive based on non-covalent inter­action plots) and π–π stacking inter­actions [inter­centroid separation = 3.9242 (9) Å]. An analysis of the Hirshfeld surface shows the important contributions made by H⋯H (35.9%) and N⋯H (26.2%) contacts to the overall surface, as well as notable contributions by O⋯H (9.9%), C⋯H (8.7%), C⋯C (7.3%) and C⋯N (7.2%) contacts.

## Abstract 2

The title mol­ecule is twisted with the dihedral angle between the N-bound 4-cyano­phenyl and C-bound acetyl groups of the 1,2,3-triazoyl ring being 60.82 (13)°. The mol­ecular packing is sustained by carbonyl-C=O⋯π(triazo­yl), cyano-C≡N⋯π(triazo­yl) and π–π stacking inter­actions.

## Chemical context   

The 1,2,3-triazoles comprise an inter­esting class of heterocyclic compounds, with diverse applications in biological and material chemistry (Struthers *et al.*, 2010 ▸; Bonandi *et al.*, 2017 ▸; Dheer *et al.*, 2017 ▸). In particular, 1,2,3-triazoles containing a carbonyl or carboxyl group in their structures have received considerable attention as they are found in a great number of biologically and pharmaceutically active mol­ecules that exhibit a broad spectrum of properties (Shu *et al.*, 2009 ▸; Morzherin *et al.*, 2011 ▸; Cheng *et al.*, 2012 ▸; Gilchrist *et al.*, 2014 ▸). In this context, the organocatalytic cyclo­addition reaction of organic azides with β-ketoesters, β-keto­amides, enones and allyl ketones has proven to be a powerful strategy for the synthesis of such class of compounds (John *et al.*, 2015 ▸; Lima *et al.*, 2015 ▸). Although much progress has been achieved, most of the available methodologies usually employ a homogenous catalyst, which can be difficult to recover. In view of environmental concerns, very recently, we reported for the first time, a heterogeneous strategy for the synthesis of 1,4,5-tris-substituted-1,2,3-triazoles through the 1,3-dipolar cyclo­addition between aryl azides and active methyl­ene compounds using CuO nanoparticles as catalyst in DMSO under microwave irradiation (Dias *et al.*, 2018 ▸). The title compound, (I), was prepared in this study and despite having been prepared by another route in a different study (Kamalraj *et al.*, 2008 ▸), no crystal structure is available. The availability of crystals in the latter study prompted the present structural analysis.
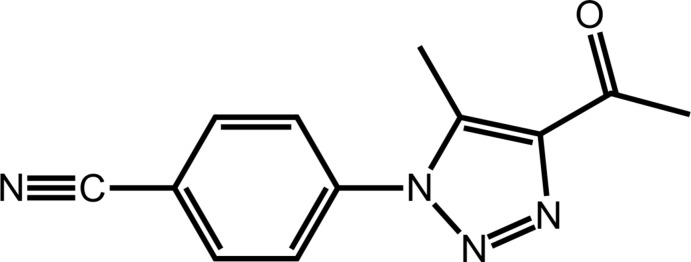


## Structural commentary   

**Figure 1:**
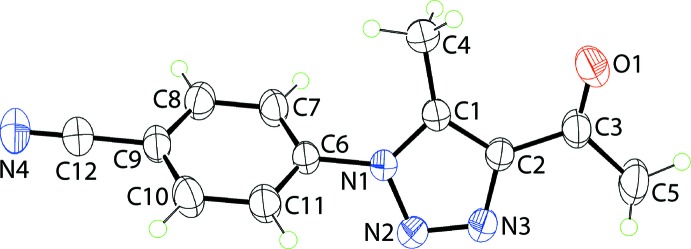
The mol­ecular structure of (I), showing the atom-labelling scheme and displacement ellipsoids at the 50% probability level.

The mol­ecular structure of (I), Fig. 1 ▸, comprises an essentially planar 1,2,3-triazolyl ring with a r.m.s. deviation of the fitted atoms of 0.0030 Å; the maximum deviation of 0.0037 (9) Å is found for the N2 atom. A 4-cyano­phenyl residue is connected to the 1,2,3-triazolyl ring at the N1-position and forms a dihedral angle of 54.64 (5)° with it, indicating a significant twist between the rings. By contrast, the acetyl group connected at the C2-position is approximately co-planar with the central ring, forming a dihedral angle of 6.8 (3)°. The dihedral angle between the phenyl and acetyl groups is 60.82 (13)°, indicating a dis-rotatory relationship. The acetyl-carbonyl group occupies a position approximately *syn* to the ring-bound methyl substituent with the C1—C2—C3—O1 and C4—C1—C2—C3 torsion angles being 6.2 (3) and −1.5 (3)°, respectively.

## Supra­molecular features   

**Figure 2:**
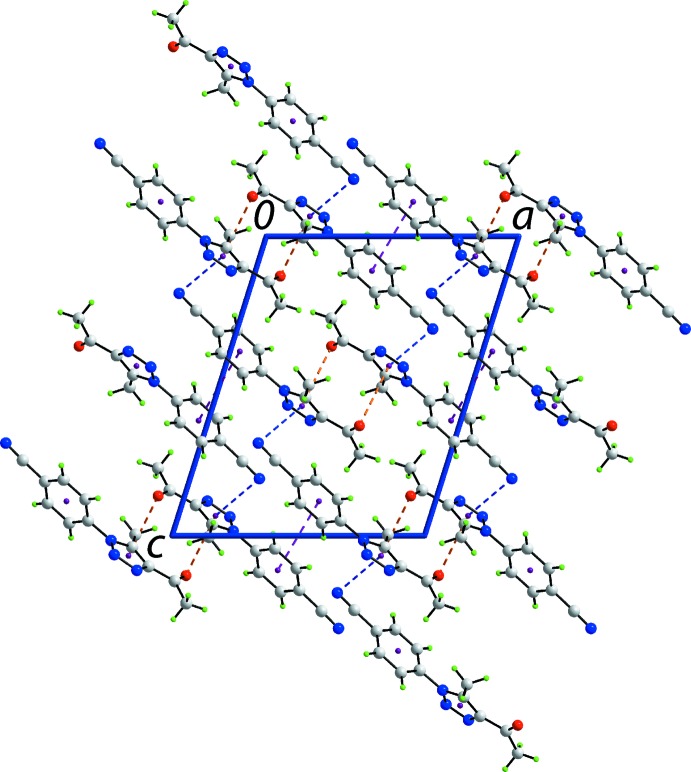
A view of the unit-cell contents shown in projection down the *b* axis. The C= O⋯π(triazo­yl), C≡N⋯π(triazo­yl) and π(tol­yl)–π(tol­yl) contacts are shown as orange, blue and purple dashed lines, respectively.

The mol­ecular packing of (I) features inter­actions involving both the five- and six-membered rings. Centrosymmetrically related mol­ecules are connected *via* carbonyl-C=O⋯π(triazo­yl) inter­actions, Table 1 ▸. Further connections between mol­ecules are of the type cyano-C≡N⋯π(triazo­yl) to the opposite face of the five-membered ring (Fig. 2 ▸, Table 1 ▸), which together lead to a supra­molecular layer parallel to (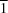
01). The O⋯π or N⋯π separations for these inter­actions are significantly longer that the van der Waals’ separations for these species (3.32 and 3.35 Å, respectively) but the non-covalent inter­actions plots (see below) indicate that they are weakly attractive in nature. Connections between the layers giving rise to a three-dimensional architecture are weak π–π stacking inter­actions between centrosymmetrically related phenyl rings, with the inter-centroid separation being 3.9242 (9) Å; symmetry operation (i): 2 − *x*, 2 − *y*, 1 − *z*. A view of the unit cell contents is shown in Fig. 2 ▸. The specified and other weak inter­molecular inter­actions are discussed in more detail below in *Hirshfeld surface analysis*.

## Hirshfeld surface analysis   

**Figure 3:**
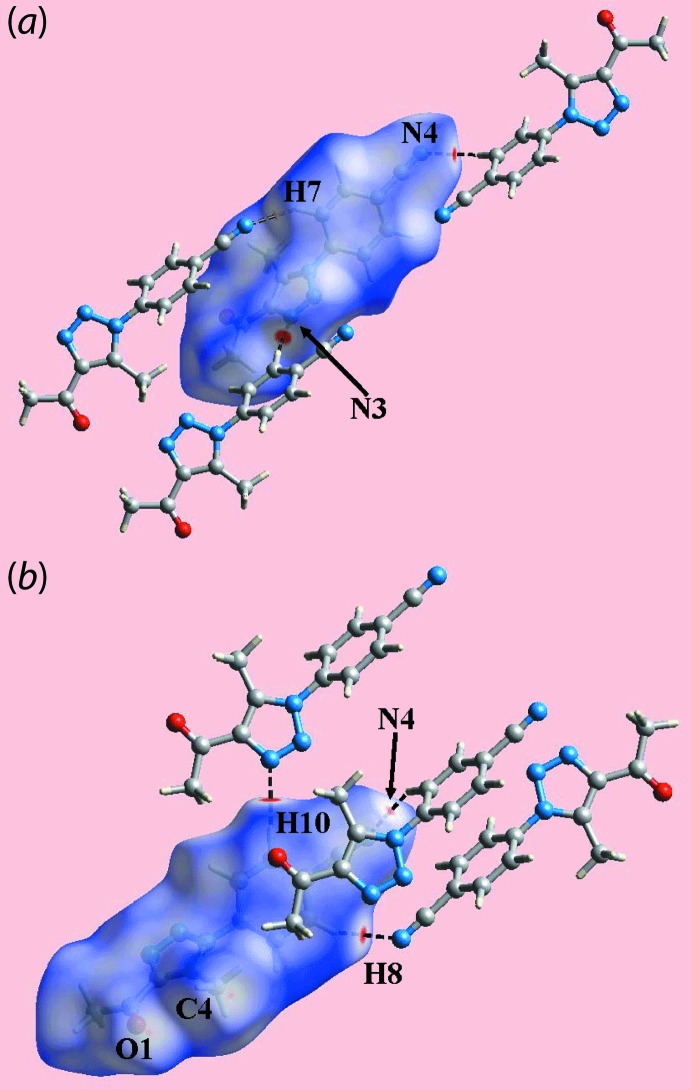
Two views of the Hirshfeld surface for (I) mapped over *d*_norm_ in the range −0.065 to +1.215 a.u.

**Figure 4:**
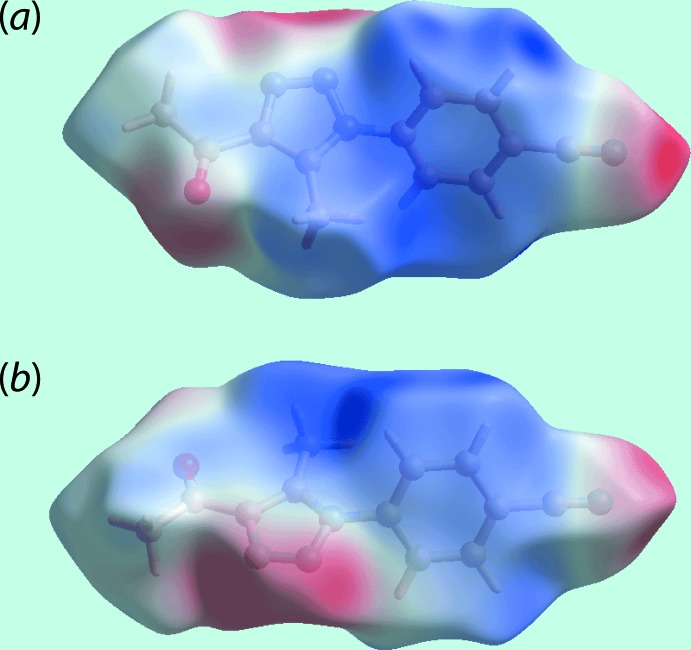
Two views of the Hirshfeld surface mapped over the electrostatic potential in the range −0.092 to +0.055 a.u. The red and blue regions represent negative and positive electrostatic potentials, respectively.

**Figure 5:**
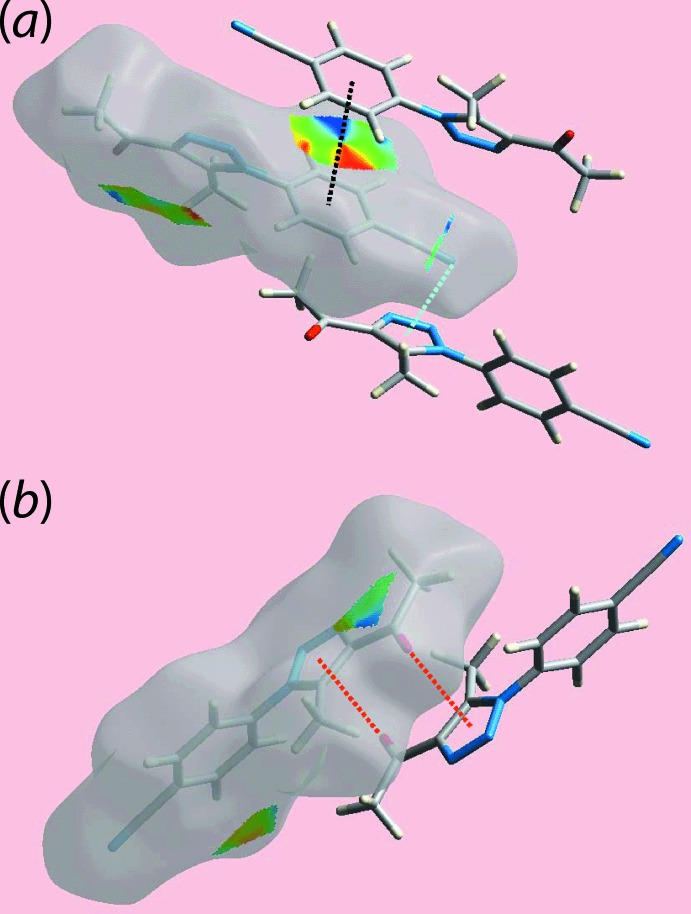
Views of the Hirshfeld surface mapped the shape-index property showing (*a*) π–π and C≡N⋯π inter­actions with black and sky-blue dotted lines, respectively and (*b*) C=O⋯π contacts with red-dotted lines.

The Hirshfeld surface calculations for (I) were performed in accord with related studies (Caracelli *et al.*, 2018 ▸) and provide information on the influence of other weak inter­molecular inter­actions instrumental in the mol­ecular packing. In addition to the presence of carbonyl-C=O⋯π(triazol­yl) and cyano-C≡N⋯π(triazol­yl) inter­actions (Table 1 ▸) in the formation of three-dimensional architecture as discussed above, the mol­ecular packing also features weak C—H⋯N inter­actions. On the Hirshfeld surface mapped over *d*_norm_ in Fig. 3 ▸, these inter­actions are characterized as the bright-red spots near the triazolyl-N3, cyano-N4 (Fig. 3 ▸ *a*), phenyl-H8 and H10 atoms (Fig. 3 ▸ *b*), and the diminutive-red spots near cyano-N4 (Fig. 3 ▸ *b*) and phenyl-H7 (Fig. 3 ▸ *a*) atoms. The influence of short inter­atomic C⋯O/O⋯C contacts involving methyl-C4 and carbonyl-O1 atoms (Table 2 ▸) is also observed as the faint-red spots near these atoms in Fig. 3 ▸ *b*. The donors and acceptors of inter­molecular C—H⋯N inter­actions are also evident as the blue and red regions corresponding to positive and negative electrostatic potentials, respectively, on the Hirshfeld surface mapped over electrostatic potential shown in Fig. 4 ▸. Views of the immediate environment about a reference mol­ecule within the Hirshfeld surface mapped over the shape-index property, highlighting inter­molecular C=O⋯π, C≡N⋯π and π–π stacking inter­actions, are illustrated in Fig. 5 ▸.

**Figure 6:**
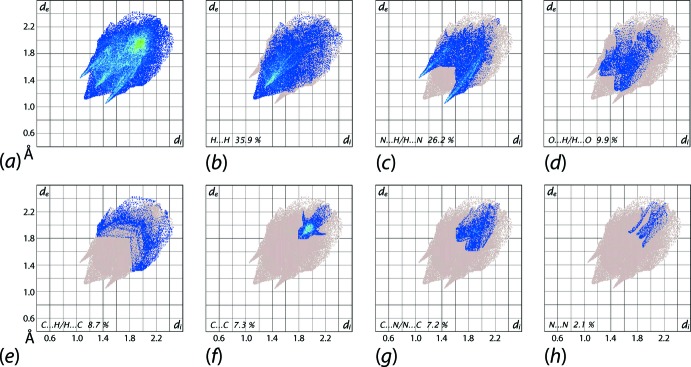
(*a*) The full two-dimensional fingerprint plot for (I) and (*b*)-(*h*) those delineated into H⋯H, N⋯H/H⋯N, O⋯H/H⋯O, C⋯H/H⋯C, C⋯C, C⋯N/N⋯C and N⋯N contacts, respectively.

The overall two-dimensional fingerprint plot for (I) (Fig. 6 ▸ *a*) and those delineated into H⋯H, N⋯H/H⋯N, O⋯H/H⋯O, C⋯H/H⋯C, C⋯C, C⋯N/N⋯C and N⋯N contacts (McKinnon *et al.*, 2007 ▸) are illustrated in Fig. 6 ▸ *b*–*i*, respectively; the percentage contributions from identified inter­atomic contacts to the Hirshfeld surface are summarized in Table 3 ▸. The short inter­atomic H⋯H contact involving symmetry-related methyl-H4*C* atoms (Table 2 ▸) is viewed as the cone-shaped tip at *d*_e_ + *d*_i_ ∼ 2.3 Å in the fingerprint plot delineated into H⋯H contacts (Fig. 6 ▸ *b*). The second largest contribution to the Hirshfeld surface, *i.e*. 26.2%, is from N⋯H/H⋯N contacts (Fig. 6 ▸ *c*) and arise from the inter­molecular C—H⋯N contacts involving cyano-N4 and triazolyl-N3 atoms (Table 2 ▸) and are viewed as the pair of overlapping green and blue spikes with their tips at *d*_e_ + *d*_i_ ∼2.5 Å. Although the carbonyl-O1 atom makes a significant contribution of 9.9% to the overall surface owing to inter­atomic O⋯H/H⋯O contacts, it is evident from the respective delineated fingerprint plot (Fig. 6 ▸ *d*) that these are beyond van der Waals separations. The relatively small contribution from C⋯H/H⋯C contacts to the Hirshfeld surface (Table 3 ▸) is indicative of the absence of C—H⋯π contacts in the mol­ecular packing, Fig. 6 ▸ *e*. The weak π–π stacking inter­actions between symmetry related phenyl-(C6–C11) rings are evident from the fingerprint delineated into C⋯C contacts (Fig. 6 ▸ *f*) as the rocket-like tip at *d*_e_ + *d*_i_ ∼ 3.6 Å. The involvement of the triazolyl ring in inter­molecular triazolyl-C≡N⋯π and carbonyl C=O⋯π contacts in the crystal is reflected from the percentage contributions due to C⋯N/N⋯C, C⋯O/O⋯C, N⋯N and N⋯O/O⋯N contacts to the Hirshfeld surface (Table 3 ▸). These inter­molecular inter­actions are also evident from the fingerprint plots delineated into C⋯N/N⋯C, C⋯O/O⋯C and N⋯N contacts in Fig. 6 ▸ *f*–*h*, respectively.

## Non-covalent inter­action plots   

**Figure 7:**
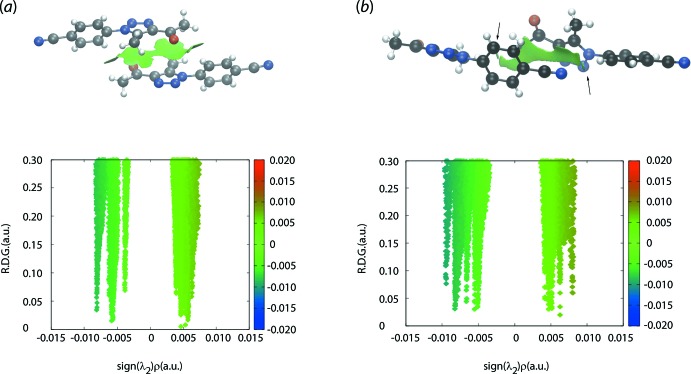
Non-covalent inter­action plots for the (*a*) carbonyl-C= O⋯π(triazol­yl) and (*b*) cyano-C≡N⋯π(triazol­yl) inter­actions. The arrows in (*b*) indicate attractive phenyl-C—H⋯N(cyano) inter­actions (see text).

Non-covalent inter­action (NCI) plots are a convenient means by which the nature of an inter­action between residues may be assessed in terms of being attractive or otherwise (Johnson *et al.*, 2010 ▸; Contreras-García *et al.*, 2011 ▸). In NCI plots, a weakly attractive inter­action will appear green on the isosurface, whereas attractive and repulsive inter­actions will result in blue and red isosurfaces, respectively. The NCI plots for the inter­acting entities of the carbonyl-C=O⋯π(triazol­yl) and cyano-C≡N⋯π(triazol­yl) inter­actions are shown in Fig. 7 ▸ *a*,*b*, indicating the weakly attractive nature of these inter­actions. The arrows in Fig. 7 ▸ *b*, highlight a weak phenyl-C—H⋯N(cyano) inter­action (Table 2 ▸).

## Database survey   

**Figure 8:**
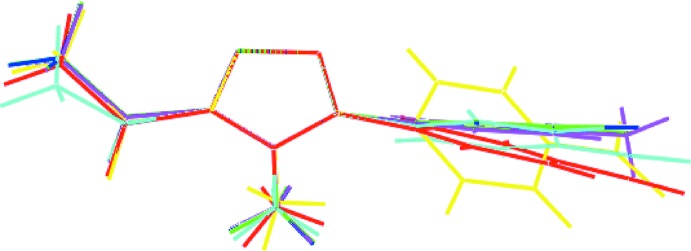
Overlay diagram for (I) and 4-*X*-phenyl derivatives: (I) (red image), *X* = Cl (green), *X* = Br (blue), *X* = Me (pink), *X* = NO_2_ (first independent mol­ecule; aqua) and *X* = NO_2_ (second mol­ecule; yellow). The mol­ecules have been overlapped so that the triazolyl rings are coincident.

There are four closely related compounds in the literature whereby the cyano group of (I) is replaced by chloride and bromide, which are isostructural (Zeghada *et al.*, 2011 ▸), methyl (El-Hiti *et al.*, 2017 ▸) and nitro (Vinutha *et al.* (2013 ▸); two independent mol­ecules comprise the asymmetric unit of the nitro compound. Key dihedral angle data are included in Table 4 ▸. This shows that the greatest variations in dihedral angles between the phenyl and acetyl residues is found for the two independent mol­ecules of the nitro compound. The different relative conformations in the aforementioned mol­ecules is highlighted in the overlay diagram of Fig. 8 ▸.

## Synthesis and crystallization   

Compound (I) was prepared as described in the literature (Dias *et al.*, 2018 ▸) and crystals were obtained by the slow evaporation from its ethyl acetate/hexane (*v*/*v*) solution. M.p. 426–428 K. ^1^H NMR (400 MHz, CDCl_3_) δ = 7.91 (*d*, *J* = 8.7 Hz, 2H), 7.65 (*d*, *J* = 8.7 Hz, 2H), 2.76 (*s*, 3H), 2.66 (*s*, 3H). ^13^C NMR (100 MHz,CDCl_3_) δ = 194.30, 144.20, 138.89, 137.42, 133.85, 125.84, 117.51, 114.23, 28.10, 10.43 ppm.

## Refinement details   

Crystal data, data collection and structure refinement details are summarized in Table 5 ▸. The carbon-bound H atoms were placed in calculated positions (C—H = 0.93–0.96 Å) and were included in the refinement in the riding model approximation, with *U*_iso_(H) set to 1.2–1.5*U*_eq_(C).

## Supplementary Material

Crystal structure: contains datablock(s) I, global. DOI: 10.1107/S2056989018010885/hb7764sup1.cif

Structure factors: contains datablock(s) I. DOI: 10.1107/S2056989018010885/hb7764Isup2.hkl

Supporting information file. DOI: 10.1107/S2056989018010885/hb7764Isup3.cml

CCDC reference: 1859008

Additional supporting information:  crystallographic information; 3D view; checkCIF report

## Figures and Tables

**Table 1:** π(Triazol­yl) inter­action geometry (Å, °) *Cg*1 is the centroid of the N1–N3/C1/C2 ring.

*D*—H⋯*A*	*D*—H	H⋯*A*	*D*⋯*A*	*D*—H⋯*A*
C3—O1⋯*Cg*1^i^	1.21 (1)	3.69 (1)	3.7359 (17)	83 (1)
C12—N4⋯*Cg*1^ii^	1.14 (1)	3.68 (1)	3.8468 (19)	90 (1)

Symmetry codes: (i) 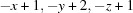; (ii) 
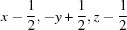
.

**Table 2:** Summary of short inter­atomic contacts (Å) in (I)

Contact	Distance	Symmetry operation
H4*C*⋯H4*C*	2.39	1 − *x*, 1 − *y*, 1 − *z*
H10⋯N3	2.48	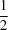 + *x*, 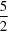 − *y*, 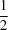 + *z*
H7⋯N4	2.58	− 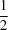 + *x*, 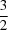 − *y*, − 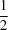 + *z*
H8⋯N4	2.53	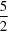 − *x*, − 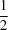 + *y*, 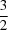 − *z*
C4⋯O1	3.208 (2)	1 − *x*, 1 − *y*, 1 − *z*

**Table 3:** Percentage contributions of inter­atomic contacts to the Hirshfeld surface for (I)

Contact	Percentage contribution
H⋯H	35.9
N⋯H/H⋯N	26.2
O⋯H/H⋯O	9.9
C⋯H/H⋯C	8.7
C⋯C	7.3
C⋯N/N⋯C	7.2
N⋯N	2.1
C⋯O/O⋯C	1.4
N⋯O/O⋯N	1.4

**Table 4:** Dihedral angle data (°) for (I) and 4-*X*-phenyl derivatives

*X*	triazol­yl/phen­yl	triazol­yl/acet­yl	phen­yl/acet­yl	Ref.
Me	50.11 (7)	6.12 (18)	50.14 (12)	El-Hiti *et al.* (2017 ▸)
Cl	45.60 (4)	6.97 (9)	45.19 (6)	Zeghada *et al.* (2011 ▸)
Br	47.03 (5)	7.08 (12)	46.5 (7)	Zeghada *et al.* (2011 ▸)
NO_2_^*a*^	38.26 (15)	13.4 (4)	27.9 (3)	Vinutha *et al.* (2013 ▸)
	87.11 (18)	15.2 (3)	74.4 (2)	
C≡N	54.64 (5)	6.8 (3)	60.82 (13)	This work

Note: (*a*) Two independent mol­ecules comprise the asymmetric unit.

**Table 5:** Experimental details

Crystal data	
Chemical formula	C_12_H_10_N_4_O
*M*_r_	226.24
Crystal system, space group	Monoclinic, *P*2_1_/*n*
Temperature (K)	293
*a*, *b*, *c* (Å)	11.8533 (5), 6.8299 (3), 14.7329 (6)
β (°)	107.477 (1)
*V* (Å^3^)	1137.67 (8)
*Z*	4
Radiation type	Mo *K*α
μ (mm^−1^)	0.09
Crystal size (mm)	0.44 × 0.27 × 0.12

Data collection	
Diffractometer	Bruker APEXII CCD
Absorption correction	Multi-scan (*SADABS*; Sheldrick, 1996 ▸)
*T*_min_, *T*_max_	0.726, 0.745
No. of measured, independent and observed [*I* > 2σ(*I*)] reflections	30812, 2333, 2083
*R*_int_	0.023
(sin θ/λ)_max_ (Å^−1^)	0.625

Refinement	
*R*[*F* ^2^ > 2σ(*F* ^2^)], *wR*(*F* ^2^), *S*	0.044, 0.126, 1.10
No. of reflections	2333
No. of parameters	156
H-atom treatment	H-atom parameters constrained
Δρ_max_, Δρ_min_ (e Å^−3^)	0.21, −0.20

Computer programs: *APEX2* and *SAINT* (Bruker, 2009 ▸), *SIR2014* (Burla *et al.*, 2015 ▸), *SHELXL2014* (Sheldrick, 2015 ▸), *ORTEP-3 for Windows* (Farrugia, 2012 ▸), *DIAMOND* (Brandenburg, 2006 ▸), *MarvinSketch* (ChemAxon, 2010 ▸) and *publCIF* (Westrip, 2010 ▸).

## supplementary crystallographic information

### Crystal data

**Table:**

C_12_H_10_N_4_O	*F*(000) = 472
*M**_r_* = 226.24	*D*_x_ = 1.321 Mg m^−^^3^
Monoclinic, *P*2_1_/*n*	Mo *K*α radiation, λ = 0.71073 Å
*a* = 11.8533 (5) Å	Cell parameters from 9978 reflections
*b* = 6.8299 (3) Å	θ = 2.6–26.3°
*c* = 14.7329 (6) Å	µ = 0.09 mm^−^^1^
β = 107.477 (1)°	*T* = 293 K
*V* = 1137.67 (8) Å^3^	Irregular, colourless
*Z* = 4	0.44 × 0.27 × 0.12 mm

### Data collection

**Table:**

Bruker APEXII CCD diffractometer	2083 reflections with *I* > 2σ(*I*)
φ and ω scans	*R*_int_ = 0.023
Absorption correction: multi-scan (SADABS; Sheldrick, 1996)	θ_max_ = 26.4°, θ_min_ = 1.9°
*T*_min_ = 0.726, *T*_max_ = 0.745	*h* = −14→14
30812 measured reflections	*k* = −8→8
2333 independent reflections	*l* = −18→18

### Refinement

**Table:**

Refinement on *F*^2^	Primary atom site location: structure-invariant direct methods
Least-squares matrix: full	Hydrogen site location: inferred from neighbouring sites
*R*[*F*^2^ > 2σ(*F*^2^)] = 0.044	H-atom parameters constrained
*wR*(*F*^2^) = 0.126	*w* = 1/[σ^2^(*F*_o_^2^) + (0.0573*P*)^2^ + 0.3916*P*] where *P* = (*F*_o_^2^ + 2*F*_c_^2^)/3
*S* = 1.10	(Δ/σ)_max_ < 0.001
2333 reflections	Δρ_max_ = 0.21 e Å^−^^3^
156 parameters	Δρ_min_ = −0.20 e Å^−^^3^
0 restraints	

### Special details

**Table:**

Geometry. All esds (except the esd in the dihedral angle between two l.s. planes) are estimated using the full covariance matrix. The cell esds are taken into account individually in the estimation of esds in distances, angles and torsion angles; correlations between esds in cell parameters are only used when they are defined by crystal symmetry. An approximate (isotropic) treatment of cell esds is used for estimating esds involving l.s. planes.

### Fractional atomic coordinates and isotropic or equivalent isotropic displacement parameters (Å^2^)

**Table:**

	*x*	*y*	*z*	*U*_iso_*/*U*_eq_	
O1	0.39515 (11)	0.7197 (2)	0.36712 (11)	0.0785 (5)	
N1	0.73587 (10)	0.95924 (17)	0.48424 (8)	0.0357 (3)	
N2	0.70535 (11)	1.13354 (19)	0.43684 (10)	0.0476 (3)	
N3	0.59641 (11)	1.11608 (19)	0.38456 (10)	0.0462 (3)	
N4	1.27442 (13)	0.8725 (3)	0.80947 (11)	0.0623 (4)	
C1	0.64418 (11)	0.8326 (2)	0.46207 (10)	0.0364 (3)	
C2	0.55549 (12)	0.9350 (2)	0.39775 (10)	0.0364 (3)	
C3	0.43345 (13)	0.8728 (2)	0.34833 (11)	0.0451 (4)	
C4	0.64687 (15)	0.6346 (3)	0.50374 (14)	0.0614 (5)	
H4A	0.7168	0.6210	0.5570	0.092*	
H4B	0.6473	0.5378	0.4566	0.092*	
H4C	0.5782	0.6168	0.5246	0.092*	
C5	0.35961 (15)	1.0069 (3)	0.27426 (14)	0.0668 (6)	
H5A	0.3902	1.0108	0.2209	0.100*	
H5B	0.3615	1.1361	0.3004	0.100*	
H5C	0.2795	0.9602	0.2538	0.100*	
C6	0.85161 (11)	0.9389 (2)	0.55064 (9)	0.0354 (3)	
C7	0.92321 (13)	0.7849 (2)	0.54291 (11)	0.0454 (4)	
H7	0.8976	0.6944	0.4938	0.054*	
C8	1.03376 (14)	0.7662 (2)	0.60904 (11)	0.0482 (4)	
H8	1.0827	0.6622	0.6050	0.058*	
C9	1.07120 (12)	0.9032 (2)	0.68124 (10)	0.0403 (3)	
C10	0.99970 (13)	1.0600 (3)	0.68695 (11)	0.0494 (4)	
H10	1.0261	1.1532	0.7347	0.059*	
C11	0.88901 (13)	1.0776 (2)	0.62138 (11)	0.0472 (4)	
H11	0.8401	1.1820	0.6249	0.057*	
C12	1.18561 (13)	0.8846 (3)	0.75178 (11)	0.0470 (4)	

### Atomic displacement parameters (Å^2^)

**Table:**

	*U*^11^	*U*^22^	*U*^33^	*U*^12^	*U*^13^	*U*^23^
O1	0.0477 (7)	0.0734 (9)	0.0927 (10)	−0.0232 (7)	−0.0117 (7)	0.0290 (8)
N1	0.0288 (6)	0.0351 (6)	0.0380 (6)	−0.0012 (4)	0.0023 (5)	0.0037 (5)
N2	0.0366 (6)	0.0393 (7)	0.0589 (8)	−0.0023 (5)	0.0022 (6)	0.0124 (6)
N3	0.0334 (6)	0.0443 (7)	0.0534 (7)	0.0003 (5)	0.0020 (5)	0.0125 (6)
N4	0.0443 (8)	0.0714 (11)	0.0552 (9)	0.0006 (7)	−0.0094 (7)	−0.0012 (7)
C1	0.0307 (6)	0.0376 (7)	0.0364 (7)	−0.0032 (5)	0.0036 (5)	0.0021 (6)
C2	0.0302 (7)	0.0401 (7)	0.0361 (7)	−0.0003 (5)	0.0054 (5)	0.0049 (6)
C3	0.0317 (7)	0.0553 (9)	0.0424 (8)	−0.0039 (6)	0.0024 (6)	0.0068 (7)
C4	0.0469 (9)	0.0476 (10)	0.0732 (12)	−0.0105 (7)	−0.0071 (8)	0.0224 (8)
C5	0.0377 (8)	0.0836 (14)	0.0641 (11)	−0.0017 (9)	−0.0074 (8)	0.0234 (10)
C6	0.0273 (6)	0.0391 (7)	0.0358 (7)	−0.0026 (5)	0.0033 (5)	0.0015 (6)
C7	0.0376 (8)	0.0462 (8)	0.0436 (8)	0.0026 (6)	−0.0009 (6)	−0.0114 (6)
C8	0.0383 (8)	0.0475 (9)	0.0512 (9)	0.0077 (6)	0.0019 (7)	−0.0067 (7)
C9	0.0301 (7)	0.0493 (8)	0.0365 (7)	−0.0029 (6)	0.0025 (5)	0.0008 (6)
C10	0.0388 (8)	0.0544 (9)	0.0477 (8)	−0.0026 (7)	0.0018 (7)	−0.0157 (7)
C11	0.0356 (8)	0.0460 (8)	0.0538 (9)	0.0032 (6)	0.0038 (7)	−0.0120 (7)
C12	0.0385 (8)	0.0526 (9)	0.0434 (8)	−0.0017 (7)	0.0025 (7)	−0.0012 (7)

### Geometric parameters (Å, º)

**Table:**

O1—C3	1.205 (2)	C5—H5A	0.9600
N1—C1	1.3500 (17)	C5—H5B	0.9600
N1—N2	1.3726 (17)	C5—H5C	0.9600
N1—C6	1.4326 (16)	C6—C7	1.378 (2)
N2—N3	1.2952 (17)	C6—C11	1.379 (2)
N3—C2	1.3634 (19)	C7—C8	1.384 (2)
N4—C12	1.140 (2)	C7—H7	0.9300
C1—C2	1.3762 (19)	C8—C9	1.385 (2)
C1—C4	1.482 (2)	C8—H8	0.9300
C2—C3	1.4734 (19)	C9—C10	1.384 (2)
C3—C5	1.491 (2)	C9—C12	1.4452 (19)
C4—H4A	0.9600	C10—C11	1.381 (2)
C4—H4B	0.9600	C10—H10	0.9300
C4—H4C	0.9600	C11—H11	0.9300
			
C1—N1—N2	111.26 (11)	C3—C5—H5C	109.5
C1—N1—C6	129.75 (12)	H5A—C5—H5C	109.5
N2—N1—C6	118.91 (11)	H5B—C5—H5C	109.5
N3—N2—N1	106.62 (11)	C7—C6—C11	121.37 (13)
N2—N3—C2	109.42 (12)	C7—C6—N1	120.33 (12)
N1—C1—C2	103.53 (12)	C11—C6—N1	118.29 (13)
N1—C1—C4	124.68 (12)	C6—C7—C8	119.25 (14)
C2—C1—C4	131.77 (13)	C6—C7—H7	120.4
N3—C2—C1	109.16 (12)	C8—C7—H7	120.4
N3—C2—C3	121.99 (13)	C7—C8—C9	119.71 (14)
C1—C2—C3	128.84 (14)	C7—C8—H8	120.1
O1—C3—C2	121.25 (14)	C9—C8—H8	120.1
O1—C3—C5	121.51 (15)	C10—C9—C8	120.56 (13)
C2—C3—C5	117.25 (14)	C10—C9—C12	118.92 (14)
C1—C4—H4A	109.5	C8—C9—C12	120.52 (14)
C1—C4—H4B	109.5	C11—C10—C9	119.66 (14)
H4A—C4—H4B	109.5	C11—C10—H10	120.2
C1—C4—H4C	109.5	C9—C10—H10	120.2
H4A—C4—H4C	109.5	C6—C11—C10	119.42 (14)
H4B—C4—H4C	109.5	C6—C11—H11	120.3
C3—C5—H5A	109.5	C10—C11—H11	120.3
C3—C5—H5B	109.5	N4—C12—C9	177.85 (18)
H5A—C5—H5B	109.5		
			
C1—N1—N2—N3	−0.81 (17)	C1—C2—C3—C5	−173.75 (16)
C6—N1—N2—N3	−177.77 (12)	C1—N1—C6—C7	57.1 (2)
N1—N2—N3—C2	0.54 (17)	N2—N1—C6—C7	−126.64 (15)
N2—N1—C1—C2	0.72 (16)	C1—N1—C6—C11	−123.36 (17)
C6—N1—C1—C2	177.26 (13)	N2—N1—C6—C11	52.95 (19)
N2—N1—C1—C4	−177.72 (16)	C11—C6—C7—C8	1.7 (2)
C6—N1—C1—C4	−1.2 (2)	N1—C6—C7—C8	−178.73 (14)
N2—N3—C2—C1	−0.10 (18)	C6—C7—C8—C9	−0.6 (3)
N2—N3—C2—C3	179.34 (14)	C7—C8—C9—C10	−1.0 (3)
N1—C1—C2—N3	−0.38 (16)	C7—C8—C9—C12	179.08 (15)
C4—C1—C2—N3	177.90 (17)	C8—C9—C10—C11	1.5 (3)
N1—C1—C2—C3	−179.78 (14)	C12—C9—C10—C11	−178.55 (15)
C4—C1—C2—C3	−1.5 (3)	C7—C6—C11—C10	−1.2 (2)
N3—C2—C3—O1	−173.09 (17)	N1—C6—C11—C10	179.25 (14)
C1—C2—C3—O1	6.2 (3)	C9—C10—C11—C6	−0.4 (3)
N3—C2—C3—C5	6.9 (2)		

### Hydrogen-bond geometry (Å, º)

π(Triazolyl) interaction geometry (Å, °) for (I). Cg1 is the centroid of the N1–N3/C1/C2 ring.

**Table:**

*D*—H···*A*	*D*—H	H···*A*	*D*···*A*	*D*—H···*A*
C3—O1···*Cg*1^i^	1.21 (1)	3.69 (1)	3.7359 (17)	83 (1)
C12—N4···*Cg*1^ii^	1.14 (1)	3.68 (1)	3.8468 (19)	90 (1)

Symmetry codes: (i) −*x*+1, −*y*+2, −*z*+1; (ii) *x*−1/2, −*y*+1/2, *z*−1/2.
